# *Limosilactobacillus reuteri* DSM 17938 relieves inflammation, endoplasmic reticulum stress, and autophagy in hippocampus of western diet‐fed rats by modulation of systemic inflammation

**DOI:** 10.1002/biof.2082

**Published:** 2024-05-27

**Authors:** Arianna Mazzoli, Maria Stefania Spagnuolo, Francesca De Palma, Natasha Petecca, Angela Di Porzio, Valentina Barrella, Antonio Dario Troise, Rosanna Culurciello, Sabrina De Pascale, Andrea Scaloni, Gianluigi Mauriello, Susanna Iossa, Luisa Cigliano

**Affiliations:** ^1^ Department of Biology University of Naples Federico II, Complesso Universitario Monte S. Angelo Naples Italy; ^2^ Institute for the Animal Production System in the Mediterranean Environment National Research Council Portici Italy; ^3^ Department of Agricultural Sciences University of Naples Federico II Portici Italy; ^4^ NBFC, National Biodiversity Future Center Palermo Italy; ^5^ Task Force on Microbiome Studies University of Naples Federico II Portici Italy

**Keywords:** autophagy, brain, cytokines, endoplasmic reticulum stress, inflammation, probiotic, western diet

## Abstract

The consumption of western diets, high in fats and sugars, is a crucial contributor to brain molecular alterations, cognitive dysfunction and neurodegenerative diseases. Therefore, a mandatory challenge is the individuation of strategies capable of preventing diet‐induced impairment of brain physiology. A promising strategy might consist in the administration of probiotics that are known to influence brain function via the gut‐brain axis. In this study, we explored whether *Limosilactobacillus reuteri* DSM 17938 (*L. reuteri*)‐based approach can counteract diet‐induced neuroinflammation, endoplasmic reticulum stress (ERS), and autophagy in hippocampus, an area involved in learning and memory, in rat fed a high fat and fructose diet. The western diet induced a microbiota reshaping, but *L. reuteri* neither modulated this change, nor the plasma levels of short‐chain fatty acids. Interestingly, pro‐inflammatory signaling pathway activation (increased NFkB phosphorylation, raised amounts of toll‐like receptor‐4, tumor necrosis factor‐alpha, interleukin‐6, GFAP, and Haptoglobin), as well as activation of ERS (increased PERK and eif2α phosphorylation, higher C/EBP‐homologous protein amounts) and autophagy (increased beclin, P62‐sequestosome‐1, and LC3 II) was revealed in hippocampus of western diet fed rats. All these hippocampal alterations were prevented by *L. reuteri* administration, showing for the first time a neuroprotective role of this specific probiotic strain, mainly attributable to its ability to regulate western diet‐induced metabolic endotoxemia and systemic inflammation, as decreased levels of lipopolysaccharide, plasma cytokines, and adipokines were also found. Therapeutic strategies based on the use of *L. reuteri* DSM17938 could be beneficial in reversing metabolic syndrome‐mediated brain dysfunction and cognitive decline.

## Graphical abstract

This study investigates the ability of *Limosilactobacillus reuteri* DSM 17938 (*L. reuteri*) in counteracting neuroinflammation, endoplasmic reticulum stress (ERS), and autophagy in hippocampus of rats fed a high fat and fructose diet. Diet‐induced pro‐inflammatory signaling pathway activation, ERS, and autophagy were prevented by *L. reuteri* administration. The neuroprotective role of this specific probiotic strain is mainly attributable to its ability to regulate western diet‐induced metabolic endotoxemia and systemic inflammation, as decreased levels of lipopolysaccharide, plasma cytokines, and adipokines were found.

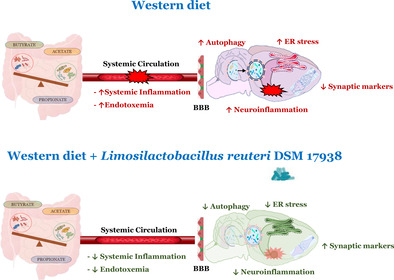

**Abbreviations**
*L. reuteri*
: *Limosilactobacillus reuteri* DSM 17938
ER: endoplasmic reticulum
ERS: endoplasmic reticulum stress
HFF: high fat high fructose
HFFR: high fat fructose supplemented with *L. reuteri*
3‐NPH: 3‐nitrophenyhydrazine
EDC: N‐(3‐dimethylaminopropyl)‐N′‐ethylcarbodiimide
LAL: Limulus amoebocyte lysate
TNF‐α: tumor necrosis factor‐alpha
IL‐6: interleukin‐6
ELISA: enzyme‐linked immunosorbent assay
BSA: bovine serum albumin
Hpt: haptoglobin
ApoE: apolipoprotein E
LC3: microtubule associated protein light chain
p62: P62‐sequestosome‐1
TLR4: toll‐like receptor‐4
NFkB: nuclear factor kappa‐light‐chain‐enhancer of activated B cells
GSK: glycogen synthase kinase 3 beta
CHOP: C/EBP‐homologous protein
PERK: protein kinase‐like endoplasmic reticulum kinase
eIF2α: eukaryotic initiation factor 2α
PSD‐95: post‐synaptic density protein 95
ZO‐1: zonula occludens‐1
IgG: immunoglobulin G
SCFAs: short‐chain fatty acids
BBB: blood brain barrier

## 1 INTRODUCTION

The consumption of Western diets, high in fats and sugars, particularly fructose, is a crucial contributor to the alarming incidence of overweight/obesity and its associated morbidities, such as type 2 diabetes mellitus,^1^, ^2^ dyslipidemia,^3^ nonalcoholic fatty liver disease,^4^ and systemic inflammation.^5^ This in turn can lead to the development of neuroinflammation, a condition often associated with depression, neurodegeneration and impaired cognitive function. Indeed, the close link between western diet‐derived metabolic dysregulation and neurodegeneration has strongly emerged in recent years, and considerable evidence has shown that western diet can impair cognition, learning and memory, both in rodents^6^, ^7^ and humans,^8^ laying the foundations for development of Alzheimer's disease.^9^

We previously reported the increase of markers of neuroinflammation in the brain of western diet‐fed rats, with the hippocampus, a brain region involved in the control of learning and memory processes, being more sensitive to the nutritional stress compared to the cortex.^10^, ^11^ In particular, the western diet regimen was shown to reduce the levels of neuronal plasticity‐related proteins in the rat hippocampus.^12^ Diet‐induced brain alterations of this importance should promote the development of novel strategies capable of limiting and/or preventing diet‐induced damage to brain physiology. In this context, an additional player in the connection between the diet and the development of neuroinflammation is the gut microbiota. In fact, it is well known that the western diet significantly alters the composition of the microbiota in the gastrointestinal tract,^13^ and that the gut microbiome can influence cognitive function via the gut‐brain axis.^14^ A strategy aimed at modulating the microbiota is based on the administration of probiotics, living microorganisms that can keep a balanced and diverse microbiota, bringing benefits to its composition and, in general, to the host health.^15^ In this regard, we have recently reported the beneficial impact of *Limosilactobacillus reuteri* DSM 17938 (*L. reuteri*) in counteracting western diet‐induced metabolic derangement in gut and liver.^16^, ^17^ This probiotic has proven to be effective in preserving the integrity of the intestinal barrier from western diet‐induced gut damage.^16^ Considering the role played by the gut‐brain axis, the aim of this study was to extend our analysis to the hippocampus of western diet‐fed rats, with the aim to explore whether novel nourishing approaches based on *L. reuteri* might be effective to counteract diet‐induced neuroinflammation, endoplasmic reticulum (ER) stress, and autophagy, also providing novel insights into the mechanism underlying its activity.

## 2 EXPERIMENTAL PROCEDURES

### 2.1 Materials

Bovine serum albumin (BSA) fraction V, nonfat milk, salts and buffers were purchased from DelTech (Naples, Italy). Fuji Super RX film, FujiFilm Man‐X Developer, and FujiFilm Man‐X Fixer were from Laboratorio Elettronico Di Precisione (Naples, Italy). Water, methanol and acetonitrile were of mass spectrometry‐grade and were obtained from Merck (Darmstadt, Germany). Along with derivatizing agents 3‐nitrophenyhydrazine (3‐NPH), *N*‐(3‐dimethylaminopropyl)‐*N*′‐ethylcarbodiimide (EDC), and quinic acid, all the analytical standards including lithium acetoacetate, sodium β‐hydroxybutyrate, and internal standards ^13^C_2_‐acetic acid, ^13^C_3_‐propionic acid, and ^13^C_4_‐butyric acid were purchased from Sigma‐Merck (Darmstadt, Germany). Pyridine was obtained from Fisher Scientific (Bremen, Germany).

### 2.2 Cultivation of *L. reuteri* DSM 17938

*L. reuteri* DSM 17938 was kindly provided by BioGaia (Noos S.r.l.; BioGaia AB, Stockholm, Sweden). It was cultured in MRS Broth (OXOID Ltd., Basingstoke, Hampshire, England) at 37°C, checked for purity and maintained on MRS Agar (Oxoid). Free cells of the strain were routinely cultured and counted on MRS Agar at 37°C for 48 h, under aerobic conditions.

### 2.3 DNA extraction, high‐throughput sequencing, and bioinformatic analysis

Fresh fecal samples of 24 rats (8 for each of the three groups) were collected after 8 weeks of treatment. DNeasy PowerSoil Pro Kit (Qiagen, Hilden, Germany) was used to extract total DNA extraction according to the manufacturer's instructions and quantified using the NanoDrop spectrophotometer. Bacterial diversity was determined by amplicon HTS of the V4‐V3 region of the 16S rRNA gene (∼460 bp). PCR and bioinformatic analysis were carried out as previously reported.^18^, ^19^, ^20^

### 2.4 Animals and treatments

All experimental procedures involving animals were approved by the “Comitato Etico‐Scientifico per la Sperimentazione Animale” of the University of Naples Federico II and were authorized by the Italian Health Ministry (137/2022‐PR). This work complies with the animal ethic principles and regulations of the Italian Health Ministry. The authors ensured that all the experimental steps were taken to minimize the pain and suffering of the animals.

Male Wistar rats (Charles River, Calco, Lecco, Italy) of 90 days were caged in a temperature‐controlled room (23 ± 1°C) with a 12 h light/dark cycle (06.30–18.30 h). The rats were divided into three groups and treated for 8 weeks with a control diet (C group; *N* = 8), or with a high fat—high fructose diet (HFF and HFFR groups; *N* = 8 for each group). In addition, HFFR rats daily received 0.5 ml of a 10% sucrose solution containing 10^8^ CFU of *L. reuteri*, while C and HFF rats received the same amount of sucrose solution without probiotics. Sucrose solution with or without probiotics was presented by an operator every day at the same hour through a needless syringe and voluntarily consumed by rats. The composition of the two diets is shown in Supplementary Table 1. At the end of the experimental period, the rats were euthanized, and hippocampus was harvested and dissected as previously described.^11^ Freshly processed aliquots were immediately snap frozen in liquid nitrogen and stored at −80°C for further analyses or fixed for immunofluorescence. Blood samples were also collected and plasma was isolated as previously reported.^12^

### 2.5 Preparation of hippocampus protein extracts

Aliquots (35 mg) of frozen hippocampus were homogenized in seven volumes of RIPA buffer (150 mM NaCl, 50 mM Tris–HCl pH 8.0, 0.5% sodium deoxycholate, 0.5% NP‐40, 0.1% SDS pH 8.0) containing 1% Protease Inhibitor Cocktail and 1% Phosphatase Inhibitor Cocktail (Euroclone, Milan, Italy). Homogenates were incubated (30 min) at 4°C and then centrifuged (14,000 g, 45 min, 4°C). Protein concentration of supernatants was measured as previously reported.^21^

### 2.6 Inflammatory parameters

Lipopolysaccharide (LPS) in plasma was measured using a protocol based on a Limulus amoebocyte lysate (LAL) extract (ThermoFisher Scientific, Rockford, IL, USA) in accordance with the manufacturer's instructions. In brief, the samples were incubated with the LAL reagent for 10 min at 37°C. Then, the chromogenic substrate solution was added for 6 min at 37°C. The reaction was stopped with a stop solution and the absorbance readings were taken on a plate reader at 405 nm.

Plasma concentrations of tumor necrosis factor‐alpha (TNF‐α), interleukin‐6 (IL‐6) were assessed using a sandwich enzyme‐linked immunosorbent assay (ELISA; R&D Systems, Minneapolis, MN, USA), specific for rats, which was in accordance with the kit instructions. Samples were diluted 1:10 and data of TNF‐α and IL‐6 were reported as ng per ml of plasma.

For quantification of TNF‐α and IL‐6 in hippocampus, proteins were extracted from slices of tissue by homogenizing frozen tissues in lysis buffer (100 mM Tris/HCl, pH 7.0, 1 M NaCl, 4 mM EDTA, 2% Triton X‐100, 0.1% sodium azide) containing 1% Protease Inhibitor Cocktail and 1% Phosphatase Inhibitor Cocktail (Euroclone, Milan, Italy). Homogenates were centrifuged at 14000 g for 30 min at 4°C and soluble samples were used for ELISA. Analysis was performed according to the manufacturer instructions in samples diluted 1:20. Data were reported as pg per mg of proteins.

Hippocampal haptoglobin (Hpt) was titrated by ELISA, in samples diluted 1: 3500; 1:7000; 1:15,000 with coating buffer (7 mM Na2CO3, 17 mM Na‐HCO3, 1.5 mM NaN3, pH 9.6), and aliquots (50 μl) were then incubated in the wells of a microtiter plate (Immuno MaxiSorp; overnight, 4°C). Washing and blocking were carried out as previously reported.^12^ Then, the wells were incubated (1 h, 37°C) with 50 μl of rabbit anti‐haptoglobin (1:500 in 130 mM NaCl, 20 mM Tris–HCl, 0.05% Tween, pH 7.4, containing 0.25% BSA), followed by 60 μl of peroxidase‐conjugated secondary antibody (1:5000 dilution). Peroxidase‐catalyzed color development from o‐phenylenediamine was measured at 492 nm.

Plasma levels of Hpt and lipocalin were assessed by Western blotting, as described below. All the plasma samples were adjusted to protein concentration of 8 μg/μl and 10 μl were used for electrophoresis on 12.5% polyacrylamide gels.^12^

### 2.7 Western blotting

Denaturing and reducing electrophoresis of hippocampal extracts^22^ or plasma proteins (30 μg or 80 μg respectively) was carried out on 12.5% polyacrylamide gels to titrate Hpt, lipocalin, apolipoprotein E (ApoE), microtubule‐associated protein light chain (LC3), P62‐sequestosome‐1 (p62), synaptophysin, synaptotagmin and IgG, or on 10% counterparts to assay toll‐like receptor‐4 (TLR4), nuclear factor kappa‐light‐chain‐enhancer of activated B cells (NFkB), beclin, glycogen synthase kinase 3 beta (GSK), C/EBP‐homologous protein (CHOP), protein kinase‐like ER kinase (PERK), eukaryotic initiation factor 2α (eIF2α), postsynaptic density protein 95 (PSD‐95), occludin, and zonula occludens‐1 (ZO‐1). Proteins blotting onto nitrocellulose membrane (GE Healthcare; Milan, Italy), washing and blocking steps were carried out according to previously published procedures.^23^, ^24^ After blocking, the membranes were incubated with primary antibodies (overnight, at 4°C), washed and then treated (1 h, at 37°C) with the appropriate peroxidase‐conjugated secondary antibodies. The specific dilution of each antibody is shown in Supplementary Table 2. As the amount of phosphorylated proteins (NFkB, GSK, PERK, eIF2α) was expressed as relative to the total NFkB, GSK, PERK, eIF2α, after revelation of the immunocomplexes, the membranes were submerged in stripping buffer (1% SDS, 25 mM glycine, pH 2; 30 min, 37°C),^22^ extensively washed, and then incubated with the specific antibody for the total form of the protein (Supplementary Table 2). For loading control, after detection of each antigen, the membranes were stripped and incubated (overnight, 4°C) with mouse anti‐β‐actin IgG (1:1000 in 0.25% v/v nonfat milk) followed by goat anti mouse‐HRP IgG (1:30000 in 0.25% v/v nonfat milk; 1 h, 37°C). Plasma Hpt and lipocalin were quantified by normalization to total protein content. In details, prior to immunodetection, membranes were stained with 0.1% Ponceau S in 5% acetic acid to determine sample loading in each lane (Supplementary Figure 1). Densitometric analysis of Ponceau staining of the membranes was then used as reference for calculating plasma protein abundance. Signal detection was carried out using the Excellent Chemiluminescent Kit Westar Antares (Cyanagen s.r.l., Bologna, Italy). Densitometric analysis of chemidoc or digital images of X‐ray films exposed to immunostained membranes was performed with Un‐Scan‐It gel software (Silk Scientific, UT, USA).

### 2.8 Immunofluorescence analysis

Paraffin embedded sections of hippocampus from all the groups were stained with the autophagy marker antibody beclin (Beclin 1 [E‐8]: sc‐48341; Santa Cruz Biotechnology), and slides were stained with DAPI (Sigma Aldrich, Saint Louis, MO, USA; diluted 1:500 in PBS). For the analysis, images were captured and visualized using Zeiss Confocal Microscope LSM 700 at 63× magnification, using a drop of immersion oil (Immersoil 518 F, Zeiss).

Three random field/section per rat were analyzed using ImageJ (National Institutes of Health, Bethesda, MD, USA).

### 2.9 Quantification of short‐chain fatty acids

Short‐chain fatty acids (SCFAs), acetate, propionate and butyrate, in rat plasma samples, were quantified according to a previous procedure,^25^ with minor modifications. Briefly, 10 μl of plasma was spiked with 1 μl of SCFAs carbon labeled internal standard mix including ^13^C_2_‐acetate, ^13^C_3_‐propionate, and ^13^C_4_‐butyrate (final concentration 0.1 mM). Plasma proteins were precipitated with the addition of 60 μl of 75% v/v methanol, while derivatization was accomplished through the mixing of suspensions with 60 μl of 200 mM 3‐NPH and 10 μl of EDC (120 mM in 6% pyridine). Upon incubation at room temperature under gentle shaking in an orbital shaker (45 min), derivatization reaction was stopped with the addition of 10 μl of 200 mM quinic acid. Samples were centrifuged at 15,000 rpm for 5 min, at 4°C, and supernatants diluted up to 1 ml with 10% v/v methanol. Samples were centrifuged again at 15,000 rpm for 5 min, at 4°C, and then analyzed without any further dilution by liquid chromatography‐high resolution mass spectrometry. Quantitation of SCFA hydrazone derivatives was achieved by a U‐HPLC system (Ultimate 3000 RS, Thermo Fisher Scientific) interfaced to a linear ion trap hybrid Orbitrap high resolution mass spectrometer (LTQ Orbitrap XL, Thermo Fisher Scientific). Mobile phases consisted of water (solvent A) and acetonitrile (solvent B), and the flow rate was 0.2 ml/min. Reversed phases chromatographic separation was achieved through the following gradient of solvent B (minutes/%B): (0/5), (5/5), (12.3/35), (13.3/85), (14/99), (16/99) by mean of a core–shell C18 column (Kinetex C18 PS, 100 × 2.1 mm, 2.6 μm; Phenomenex, Torrance, CA), thermostated at 40°C. Liquid chromatographic stream was interfaced to an electrospray ion source working in negative ion mode, scanning the ion in the *m*/*z* range 100–400; resolution was set at 30,000 (FWHM at *m*/*z* 200), capillary temperature was 300°C, while sheath and auxiliary gases were set at 25 and 15 arbitrary units, respectively. Analyte profile data in full MS mode were collected using Xcalibur 2.1 (Thermo Fisher Scientific). Calibration curve was obtained with the internal standard technique in the linearity range 0.001–1 mM. Analytical performances are detailed in Supplementary Table 3.

### 2.10 Statistical analysis

Data were expressed as mean values ± SEM. GraphPad Prism 9.3.1 (GraphPad Software, San Diego, CA, USA) was used to verify normal distribution of data and to compare groups with one‐way ANOVA followed by Bonferroni post test. *p* < 0.05 was considered significant in the reported analyses.

## 3 RESULTS

### 3.1 Hippocampal inflammation

Western diet consumption is characterized by the onset of inflammation in critical brain areas such as hippocampus, a key cerebral area for learning and memory. Therefore, the potential impact of probiotic administration in this district is of relevance. We therefore evaluated the activation of pro‐inflammatory signaling pathways by measuring the degree of NFkB phosphorylation, the amount of TLR4 and the levels of TNF‐α, IL‐6, and GFAP, to verify whether *L. reuteri*‐supplemented rats could be protected from western diet‐induced hippocampal inflammation.

**FIGURE 1:**
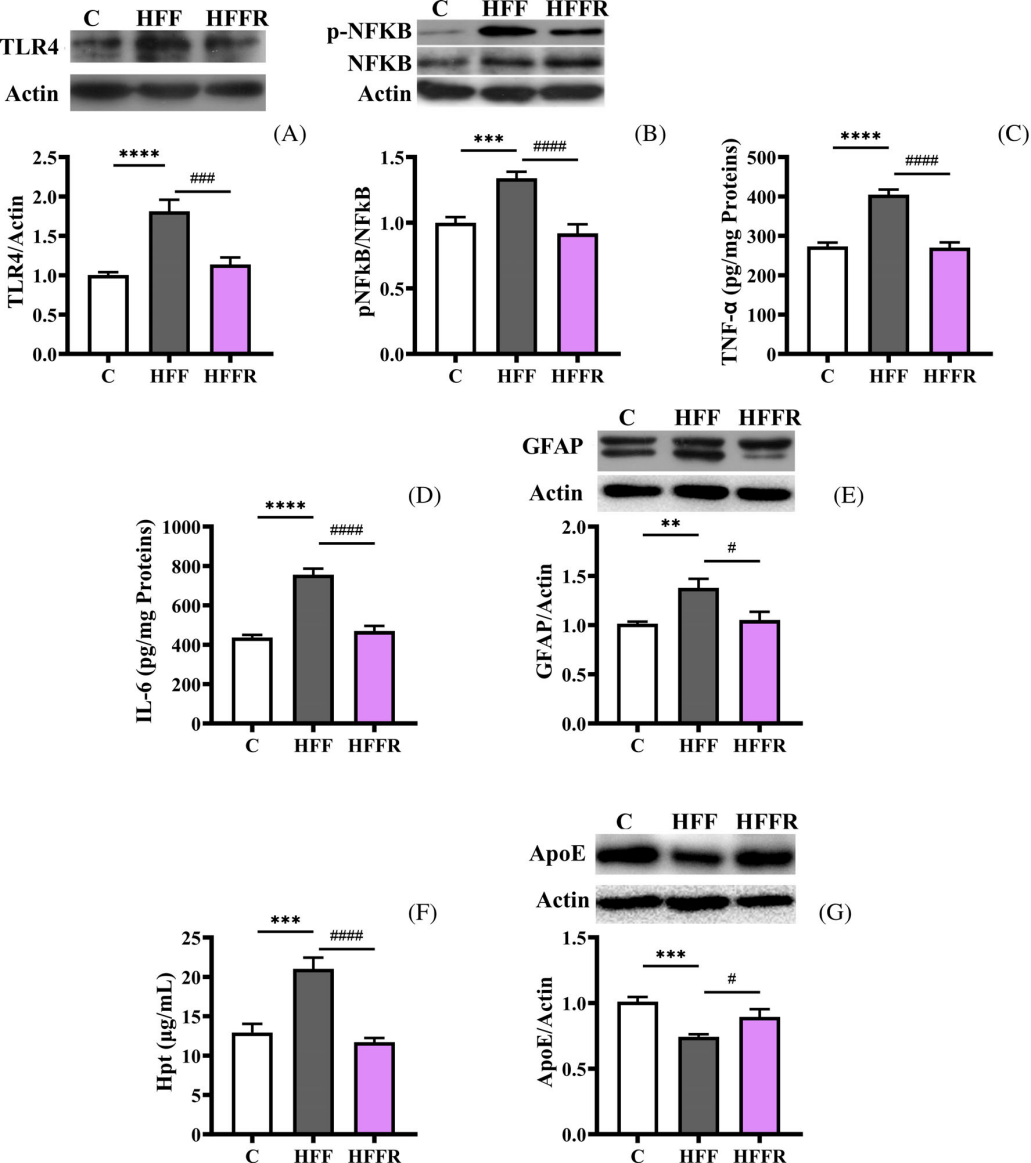
Markers of neuroinflammation. Toll‐like receptor 4 (TLR4) protein content (with representative blots, normalized to controls) (A), phosphorylated NFkB/NFkB ratio (with representative blots, normalized to controls) (B), tumor necrosis factor alpha (TNF‐α) (C) and interleukin 6 (IL‐6) (D) content, glial fibrillar acidic protein (GFAP) (with representative blots, normalized to controls) (C), haptoglobin (Hpt) (F) and apolipoprotein E (ApoE) (with representative blots, normalized to controls) (G) protein content in hippocampus from rats fed control diet (C), high fat fructose diet (HFF) and high fat fructose diet supplemented with *Limosilactobacillus reuteri* (HFFR). Values are the means ± SEM of eight different rats. **p < 0.01, ***p < 0.001, ****p < 0.0001 compared to C rats; ^#^ *p* < 0.05, ^###^ *p* < 0.001, ^####^ *p* < 0.0001 compared to HFF rats (one‐way ANOVA followed by Bonferroni posttest).

As shown in Figure 1, the western diet‐induced increase of both TLR4 amount and NFkB phosphorylation (used as a marker of the activation of NFkB) (HFF vs. C) was prevented by probiotic administration in hippocampus of HFFR rats (Figure 1A,B). In line with these results, both TNF‐α and IL‐6 concentrations were higher in hippocampus of HFF rats compared to controls, but not in HFFR rats (Figure 1C,D). Further, the amounts of GFAP, a marker of astrogliosis, and Hpt, an inflammatory marker very sensitive to nutritional stress,^12^ were higher in HFF rats respect to the control, and this rise was prevented by *L. reuteri* treatment in HFFR group (Figure 1E,F). Accordingly, the amount of ApoE, a pleiotropic protein that has been shown to reduce glial activation and brain inflammatory response in vitro and in vivo,^26^, ^27^ was found reduced in HFF rats, while the probiotic administration prevented this alteration in HFFR rats (Figure 1G).

These results show that western diet‐associated activation of pro‐inflammatory pathways can be fully prevented by the concomitant administration of *L. reuteri*.

### 3.2 Hippocampal ERS and autophagy

**FIGURE 2:**
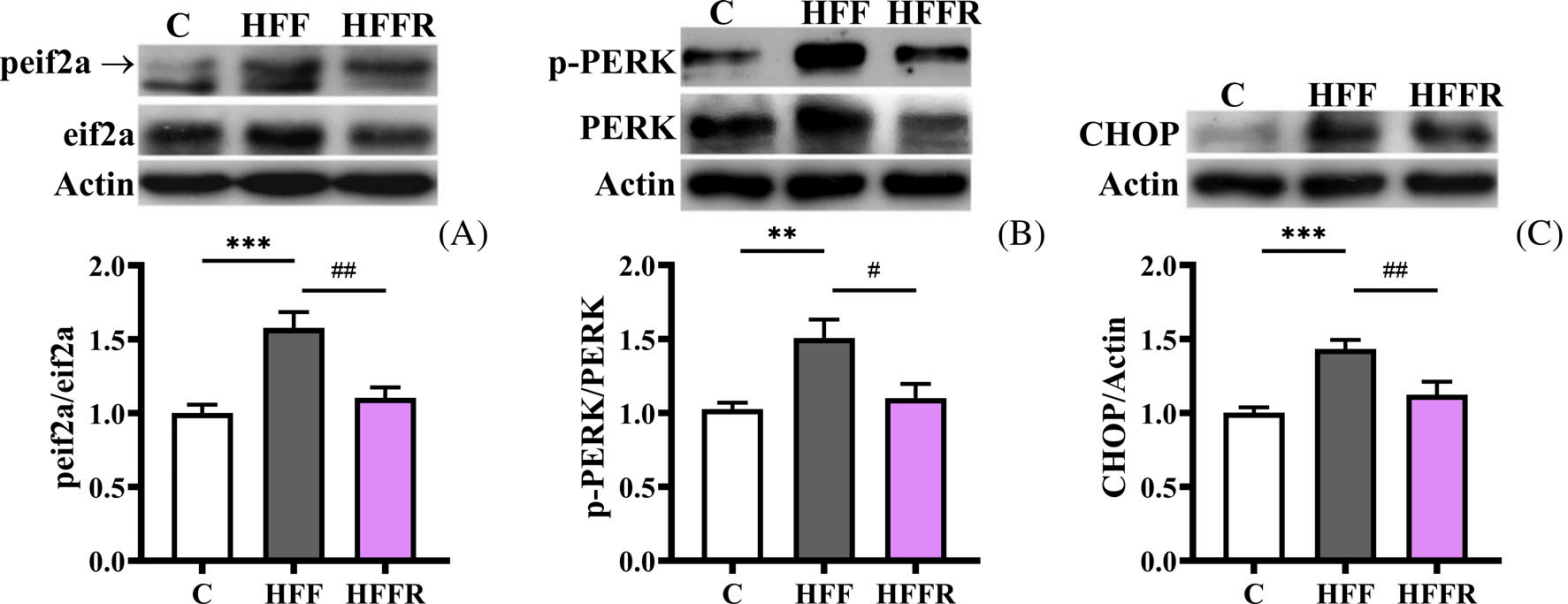
Markers of endoplasmic reticulum (ER) stress. Phosphorylated protein kinase RNA‐like ER kinase (PERK)/PERK ratio (with representative blots, normalized to controls) (A), phosphorylated eukaryotic initiation factor‐2 (pEIF2)/EIF2 ratio (with representative blots, normalized to controls) (B), C/EBP homologous protein (CHOP) content (with representative blots, normalized to controls) (C) in hippocampus from rats fed control diet (C), high fat fructose diet (HFF) and high fat fructose diet supplemented with *Limosilactobacillus reuteri* (HFFR). Values are the means ± SEM of eight different rats. **p < 0.01, ***p < 0.001 compared to C rats; ^#^ *p* < 0.05, ^##^ *p* < 0.01 compared to HFF rats (one‐way ANOVA followed by Bonferroni posttest). PERK and CHOP markers (panel B and C, respectively) were from the same membrane, so the same actin is shown as loading control.

Inflammation, leading to the production of inflammatory cytokines, could trigger ERS causing the accumulation of misfolded and unfolded proteins.^28^, ^29^ The strong interplay among metabolic dysfunction, inflammation and ERS has been previously described.^30^ In particular, excessive dietary fat or simple carbohydrate were reported to contribute to ER stress in liver or pancreas.^31^ We therefore investigated whether WD‐induced inflammation triggers the activation of ERS in hippocampus and the efficacy of *L. reuteri* in modulating this pathway. To this aim, we assessed the level of ERS indicators, including the degree of PERK and eIF2α phosphorylation, and the amount of their downstream effector, the transcription factor CHOP. Indeed, under condition of stress, the protein PERK undergoes dimerization and autophosphorylation; activated PERK phosphorylates the translation initiation factor eIF2α, which, in turn, activates the transcription of CHOP.^29^ The activation of PERK signaling pathway, as evidenced by the increased p‐PERK/PERK and p‐eIF2α/eIF2α ratios, and higher levels of CHOP was induced in HFF rats, but this alteration was prevented by the concomitant administration of *L. reuteri* in HFFR rats (Figure 2). These results revealed that western diet‐induced ERS in the hippocampus was counteracted by probiotic administration.

**FIGURE 3:**
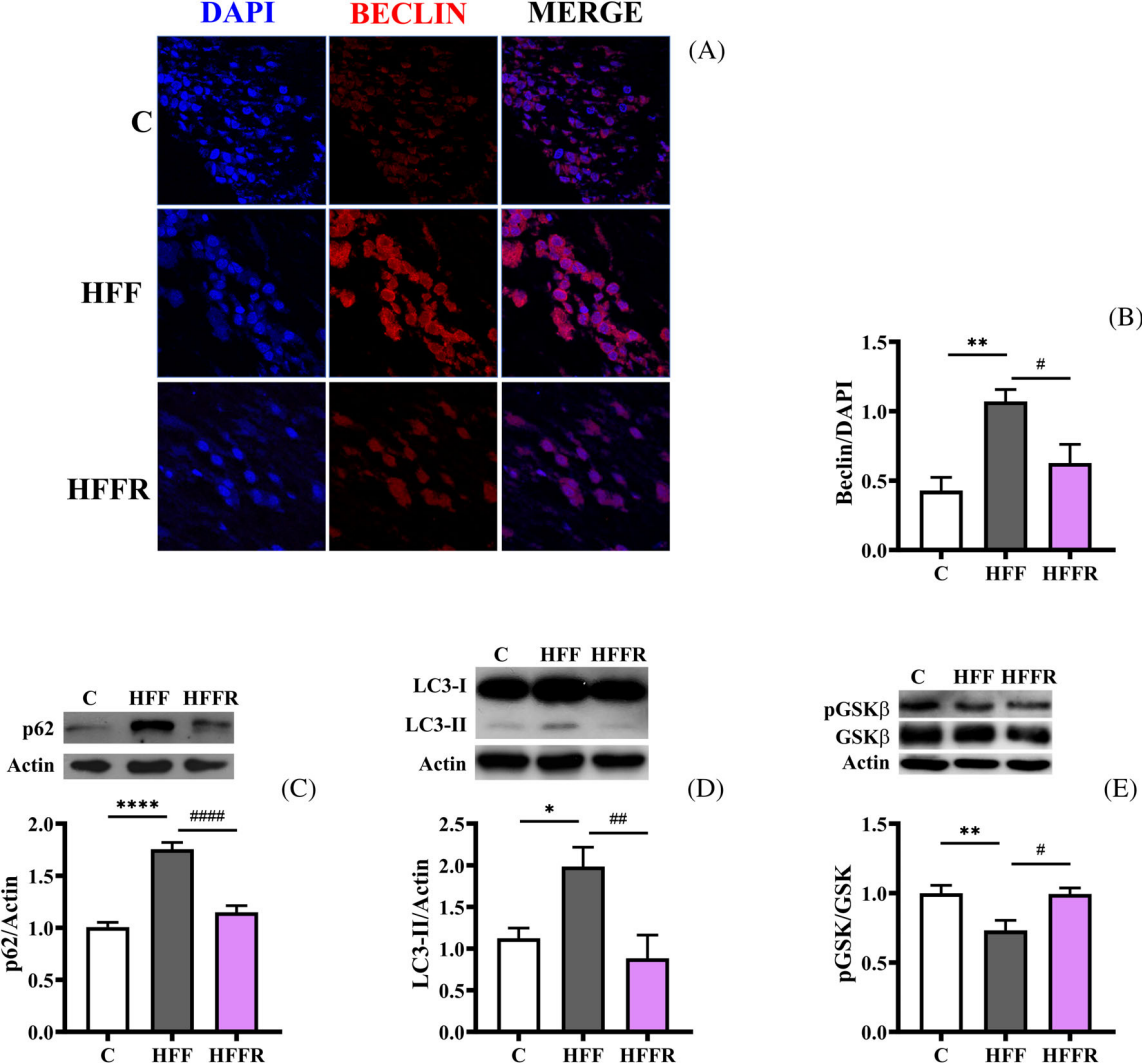
Markers of autophagy. Immunofluorescence representative images (A) with relative quantification (B) of beclin, p62 (C), and microtubule‐associated protein 1A/1B‐light chain 3 (LC3‐II) (D) protein content (with representative blots, normalized to controls), and phosphorylated glycogen synthase kinase (GSK)/GSK ratio (with representative blots, normalized to controls) (E), in hippocampus from rats fed control diet (C), high fat fructose diet (HFF) and high fat fructose diet supplemented with *Limosilactobacillus reuteri* (HFFR). Values are the means ± SEM of eight different rats. *p < 0.05, **p < 0.01, ****p < 0.0001 compared to C rats; ^#^ *p* < 0.05, ^##^ *p* < 0.01, ^####^ *p* < 0.0001 compared to HFF rats (one‐way ANOVA followed by Bonferroni posttest).

Evidence has revealed ERS response as a potential trigger for another major response pathway to cellular stress, namely autophagy.^32^, ^33^ As a matter of fact, the transcription factor CHOP drives the expression of autophagy proteins to initiate the formation of autophagosomes.^32^ We therefore investigated whether diet‐induced ERS was also associated with activation of the autophagic process in the rat hippocampus. To this aim, we assessed the levels of beclin (Figure 3A,B; Supplementary Figure 2), p62 (Figure 3C), and LC3‐II (Figure 3D). As shown in Figure 3A–D, a significant diet‐related increase of beclin, p62 and LC3‐II was observed in the hippocampus of HFF rats, and this increase was prevented by the concomitant administration of *L. reuteri* in HFFR rats. ERS is also highly intertwined with GSK pathway,^34^ whose activation was reported to be induced by ERS^35^ and is known to trigger autophagy.^36^ In line with these observations, we detected a decreased inhibitory phosphorylation of GSK, that is greater activation, in western diet‐fed rats (Figure 3E), that was prevented by *L. reuteri* treatment in HFFR rats.

These results indicated that the autophagic process was activated in the hippocampus of western diet‐fed rats, and *L. reuteri* was effective in contrasting this activation.

### 3.3 Neuronal plasticity related proteins

**FIGURE 4:**
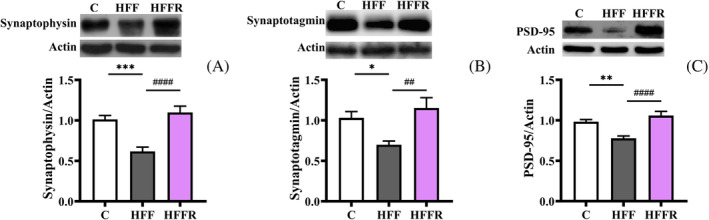
Synaptic proteins. Synaptophysin (A), synaptotagmin (B), and postsynaptic density protein 95 (PSD‐95) (C) (with representative blots, normalized to controls) in hippocampus from rats fed control diet (C), high fat fructose diet (HFF) and high fat fructose diet supplemented with *Limosilactobacillus reuteri* (HFFR). Values are the means ± SEM of eight different rats. *p < 0.05, **p < 0.01, ***p < 0.001, compared to C rats; ^##^ *p* < 0.01, ^####^ *p* < 0.0001 compared to HFF rats (one‐way ANOVA followed by Bonferroni posttest).

As both neuroinflammation and ER stress are associated with synaptic loss,^37^, ^38^, ^39^ we verified whether the protective effect of *L. reuteri* was able to preserve hippocampal expression of plasticity‐related proteins. Indeed, we observed that presynaptic and postsynaptic proteins, namely synaptophysin and synaptotagmin I (Figure 4A,B), and the postsynaptic protein PSD‐95 (Figure 4C), respectively, with a key role in synaptic plasticity,^40^ were reduced by the western diet (HFF) but were preserved by *L. reuteri* administration (HFFR).

### 3.4 Microbiota composition, SCFAs, systemic inflammation and blood–brain barrier

**FIGURE 5:**
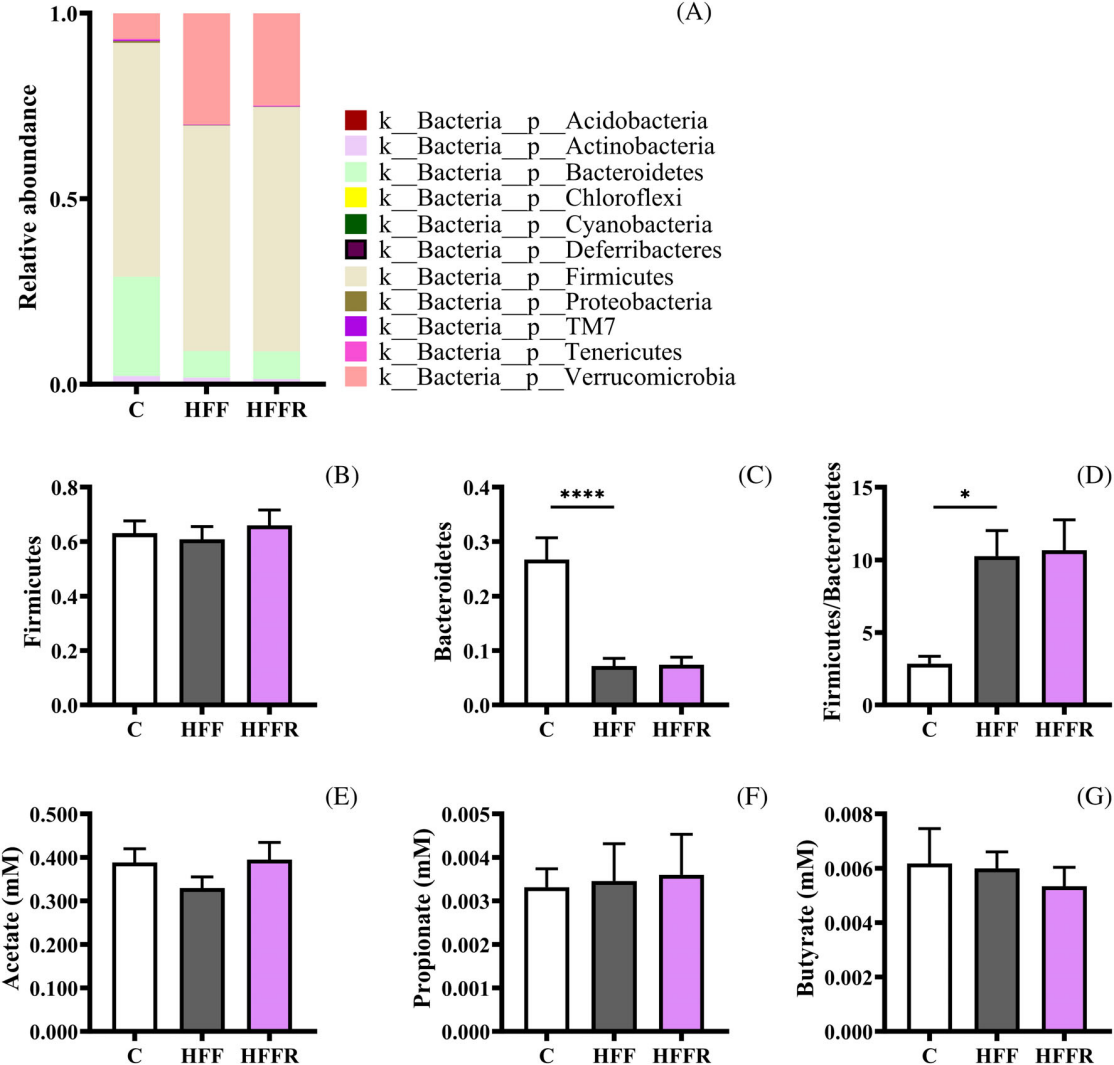
Gut microbiota and plasma short‐chain fatty acids. Bar plots showing the fecal microbial composition at the phylum level (A), Firmicutes and Bacteroidetes quantification (B,C), Firmicutes/Bacteroidetes ratio (D) plasma acetate (E), propionate (F), and butyrate (G) in rats fed control diet (C), high fat fructose diet (HFF) and high fat fructose diet supplemented with *Limosilactobacillus reuteri* (HFFR). Values are the means ± SEM of six different rats. *p < 0.05, **** *p* < 0.0001 compared to C rats (one‐way ANOVA followed by Bonferroni posttest).

In order to investigate whether the hippocampal anti‐inflammatory action of *L. reuteri* could derive from changes in gut microbiota, the specific composition of gut bacteria species was assessed. *Firmicutes*, *Bacteroidetes*, and *Verrucomicrobia* were the most abundant species in control rats (Figure 5A), in agreement with previous studies.^41^ The western diet did not modify the number of *Firmicutes* (Figure 5B) but induced a significant decrease in the number of *Bacteroidetes* (Figure 5C), with a consequent increase in the *Firmicutes/Bacteroidetes* ratio (F/B) (Figure 5D) in HFF rats, a known marker of gut dysbiosis, associated with the development of several pathologies.^42^ Of note, the probiotic administration was not able to counteract the diet effect on the gut microbiota composition. Indeed, the HFFR rats showed a microbiota profile similar to that of HFF rats (Figure 5A–D), thus ruling out the hypothesis that the brain‐protecting effect of *L. reuteri* could be mediated by the reshaping of gut microbiota.

Different evidence reported the protective effect of SCFAs on the brain, as provided by their direct action on anti‐inflammatory and anti‐oxidative cellular pathways.^43^ The main SCFAs of gut bacterial origin, namely acetate, propionate, and butyrate, were thus assessed in plasma by a dedicated liquid chromatography‐high resolution mass spectrometry procedure to evaluate their possible involvement in the protection of brain functions exerted by *L. reuteri*; no difference in plasma levels of acetate, propionate and butyrate were found between the three different groups (Figure 5E–G), emphasizing the absence of a role of SCFAs in this phenomenon.

**FIGURE 6:**
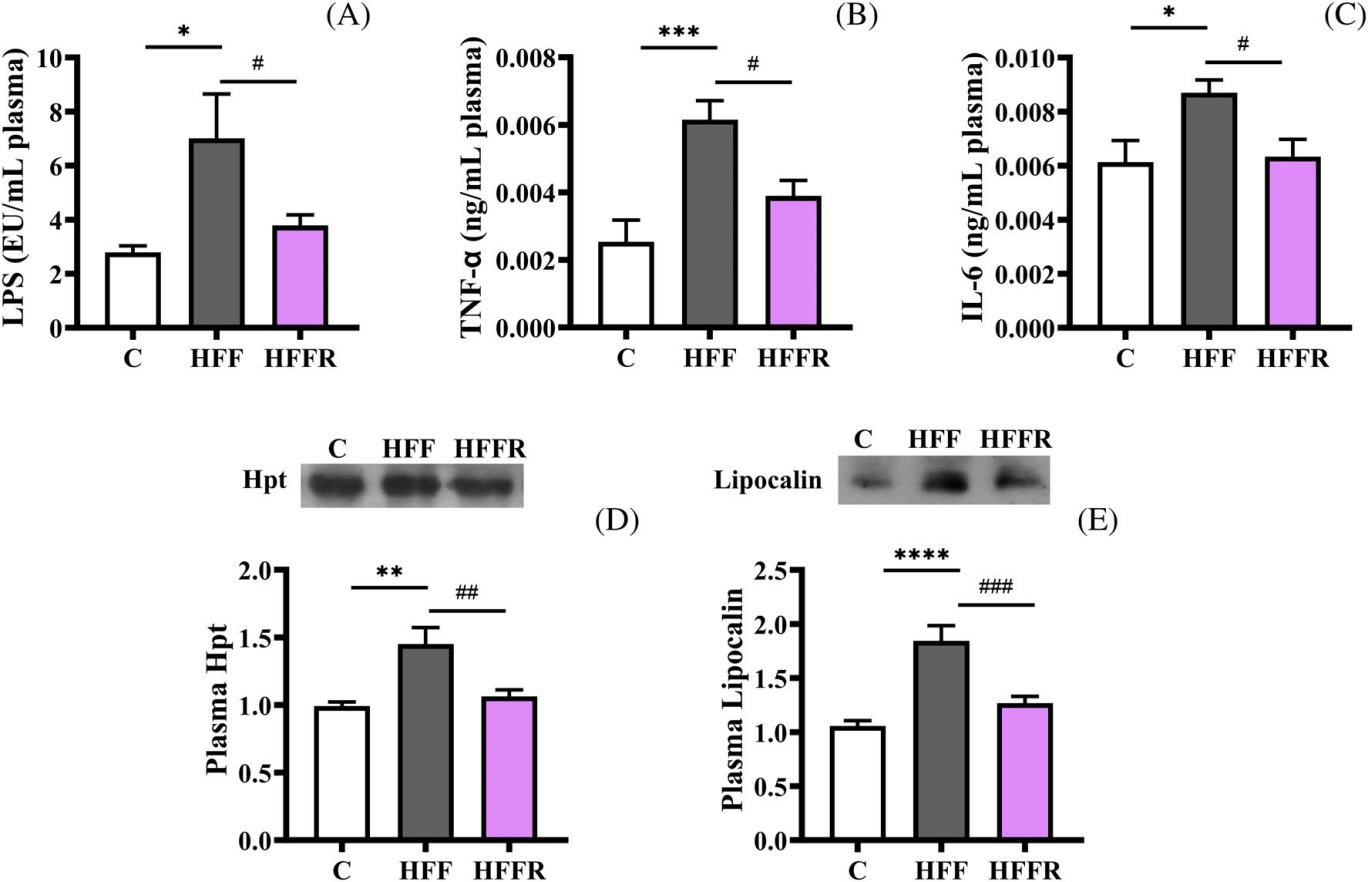
Markers of plasma inflammation. Plasma levels of lipopolysaccharide (LPS) (A), tumor necrosis factor alpha (TNF‐α) (B), interleukin 6 (IL‐6) (C), and haptoglobin (Hpt) (D) and lipocalin (E) (with representative blots, normalized to controls) protein content in hippocampus from rats fed control diet (C), high fat fructose diet (HFF) and high fat fructose diet supplemented with *Limosilactobacillus reuteri* (HFFR). Hpt and lipocalin abundance was normalized to total protein content, assessed by Ponceau S staining of the membrane prior to immunodetection. Values are the means ± SEM of eight different rats. *p < 0.05, **p < 0.01, ***p < 0.001, ****p < 0.0001 compared to C rats; ^#^ *p* < 0.05, ^##^ *p* < 0.01, ^###^ *p* < 0.001 compared to HFF rats (one‐way ANOVA followed by Bonferroni posttest).

Probiotics are generally considered to exert an anti‐inflammatory activity, with an impact not only on the gut but also on peripheral organs. In agreement, *L. reuteri* proved to be effective in avoiding, in HFFR rats, the western diet‐induced metabolic endotoxemia and systemic inflammation, characterized by increased plasma levels of LPS, of pro‐inflammatory cytokines TNF‐α, IL‐6 (Figure 6A–C), and of further inflammatory proteins Hpt and lipocalin (Figure 6D,E), observed in the HFF counterparts.

**FIGURE 7:**
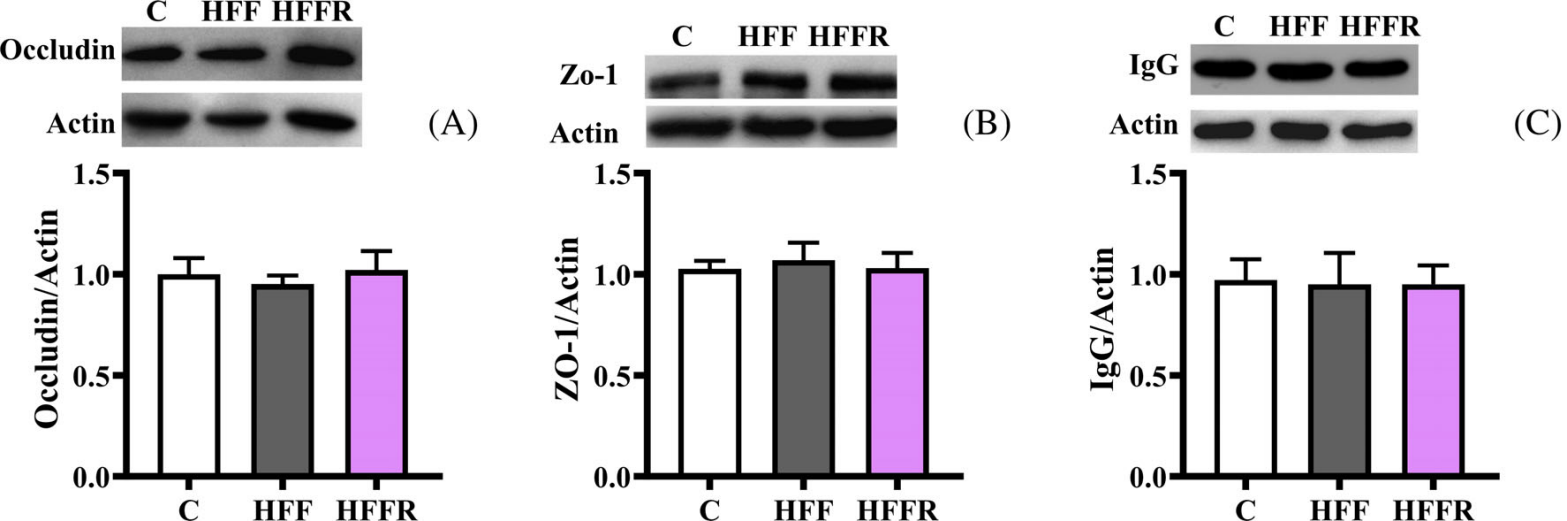
Markers of blood–brain barrier integrity. Occludin (A), ZO‐1 (B), and immunoglobulin G (IgG) protein content (with representative blots, normalized to controls) in hippocampus from rats fed control diet (C), high fat fructose diet (HFF) and high fat fructose diet supplemented with *Limosilactobacillus reuteri* (HFFR). Values are the means ± SEM of eight different rats.

Western diet‐induced systemic inflammation could impact on hippocampal inflammation due to the alteration of the blood–brain barrier (BBB). Indeed, high‐fat or cholesterol‐enriched diets have been shown to disrupt BBB.^44^, ^45^ We therefore investigated brain concentration of occludin and ZO‐1, two tight junction proteins of the BBB as well as the amount of cerebral IgG, whose leakage into the brain represents a marker of BBB permeability alteration.^46^ No diet‐induced alteration in BBB was found, as the amounts of occludin, ZO‐1, and IgG were not significantly different between control and HFF rats or HFFR rats (Figure 7A–C). These results proved that the hippocampal inflammation observed in the HFF group of rats was not related to alterations of BBB.

## 4 DISCUSSION

Several studies indicate positive health benefits of Lactobacillus supplementation on different organs,^47^ but little research has focused on the potential activity of these bacteria species in rescuing hippocampus alterations induced by a nutritional insult such as a diet enriched in fats and sugars.

We have recently reported the beneficial influence of *L. reuteri* DSM 17938 on gut and liver impairment induced by a western diet.^16^, ^17^ In this study, we extended our analysis to the hippocampus, elucidating the beneficial impact of the probiotic *L. reuteri* on neuroinflammation and related pathways which is a common stress target of a western diet regimen. In line with our previous results,^10^, ^12^ western dietary treatment induced hippocampal inflammation, as assessed by the finding of NFkB pathway activation, which is in turn responsible of the increased levels of key inflammatory cytokines namely TNF‐α and IL‐6, as well as of glial activation, evidenced by enhanced levels of GFAP. Our analysis was also extended to protein players with key role not only in modulation of inflammation but also in the regulation of oxidative homeostasis^48^ or cholesterol metabolism,^10^ namely Hpt and ApoE, respectively. The probiotic administration was able to prevent the rise of inflammatory process, in agreement with a previous report showing the efficacy of another lactobacillus against brain inflammation induced by a western diet.^49^

Moreover, inflammation can also trigger ERS,^28^, ^29^ which in turn activates downstream signaling pathways leading to NFkB activation, with consequent amplification of the inflammatory response.^32^ As a matter of fact, we observed an increased degree of phosphorylation of PERK, which is indicative of a condition of ERS^31^ in hippocampus of HFF rats. Further, the activation of PERK pathway was associated with higher phosphorylation of its target protein elF2α, as well as by higher levels of the transcription factor CHOP in HFF rats. Interestingly, we also detected significantly higher levels of the autophagy‐related proteins beclin, p62 and LC3‐II in HFF fed rats respect to control animals. The activation of autophagy response, which represents a major intracellular degradation system,^32^, ^33^ is suggestive of a strong impairment of ER homeostasis and function.^32^ In this frame, it is worth mentioning that ERS and neuroinflammation influence neuronal physiology, as they are associated with synaptic loss, leading to altered neuronal plasticity and behavior.^38^, ^39^ Indeed, chronic PERK signaling is involved in the repression of the expression of a synaptic proteins cluster.^38^ Accordingly, we found decreased levels of synaptophysin, synaptotagmin, and the postsynaptic protein PSD‐95 in the hippocampus of HFF rats, confirming that the western diet, through the neuroinflammatory processes, compromises synaptic function and neural viability. Although unbalanced diets and obesity were previously reported to be associated with ERS,^50^, ^51^, ^52^ to our knowledge this is the first study showing the effects of a western diet on the integrated activation of the major response pathways related to stress in rat hippocampus and, more importantly, the ability of *L. reuteri* DSM 17938 to avoid diet‐induced inflammation as well as ERS and autophagy.

The probiotic treatment in western diet fed animals can impact health and wellbeing through different routes. Considering the efficacy of *L. reuteri* in protecting the hippocampus from the western diet‐induced alterations, several hypotheses can be formulated about its mechanism of action in our experimental paradigm. Given the potential effect of a probiotic administration on gut microbiota composition and/or on metabolites of bacterial origin such as SCFAs, our first speculation was that *L. reuteri* could have modulated gut microbiota composition and/or production of SCFAs. From the analysis of the microbiota, evidencing a condition of gut dysbiosis induced by the western diet and not reverted by the probiotic administration, we can discard the hypothesis that enrichment in specific bacterial populations is involved in the beneficial effect of *L. reuteri*. We then explored the possibility that the *L. reuteri* administration was associated with changes of SCFAs level, but we did not detect significant differences in serum levels of these compounds in western diet‐fed rats treated with *L reuteri* rats compared to rats fed only the western diet. This is interesting, because SCFAs, deriving from the fermentation of indigestible fibers and represented mostly by butyrate, propionate, and acetate,^43^ are known to have anti‐inflammatory properties^43^, ^53^ and Lactobacillus is among the microbiota species often related to increased levels of SCFAs.^53^ These results let us to rule out that changes in SCFAs levels are involved in the beneficial effect of *L. reuteri* in our experimental model of diet‐induced metabolic syndrome.

Although our results seem to be in contrast with a large part of the scientific literature that mainly focuses on the anti‐inflammatory effects of probiotics in relation to the induced change of SCFAs level, in the context of the gut‐brain axis, this study well agree with reports evidencing that the probiotic efficacy and the underlying mechanism can be highly dependent on experimental diets used as well as the specific *Lactobacillus* strain.^54^

An alternative route of action arises from the fact that the dysbiosis causes the development of endotoxemia, due to an increase in intestinal barrier permeability induced by the western diet that let the LPS produced by Gram‐negative bacteria to pass into the bloodstream, provoking low grade chronic inflammation.^55^ This systemic inflammation in turn can impact on brain, because the increased concentration of molecular mediators of inflammation (TNF‐α or IL‐6) in systemic blood drives their increase also in the brain.^56^ Our data evidenced that *L. reuteri* proved to be effective in preventing western diet‐induced metabolic endotoxemia, by decreasing the level of LPS and systemic inflammation, as reduced levels of cytokines and adipokines were observed, while we did not find changes in the levels of occludin and ZO‐1, as well as in cerebral IgG, known markers of the integrity of BBB, so allowing us to exclude that alteration of the BBB integrity is involved in the mechanism of action of *L. reuteri*.

In the light of the above consideration, the scenario that emerges from our data is that the lactobacillus strain *L. reuteri* DSM 17938 may have its impact on hippocampus regardless of its effect on the intestinal bacterial populations or on the production of SCFAs. Indeed, the results show that the beneficial impact on the brain may directly depend on the probiotic‐induced action on the health of the intestinal barrier^16^ and reduced level of LPS translocated into the blood. This in turn is reflected in the reduced production of circulating inflammatory mediators and their reduced passage in the hippocampal area.

**FIGURE 8:**
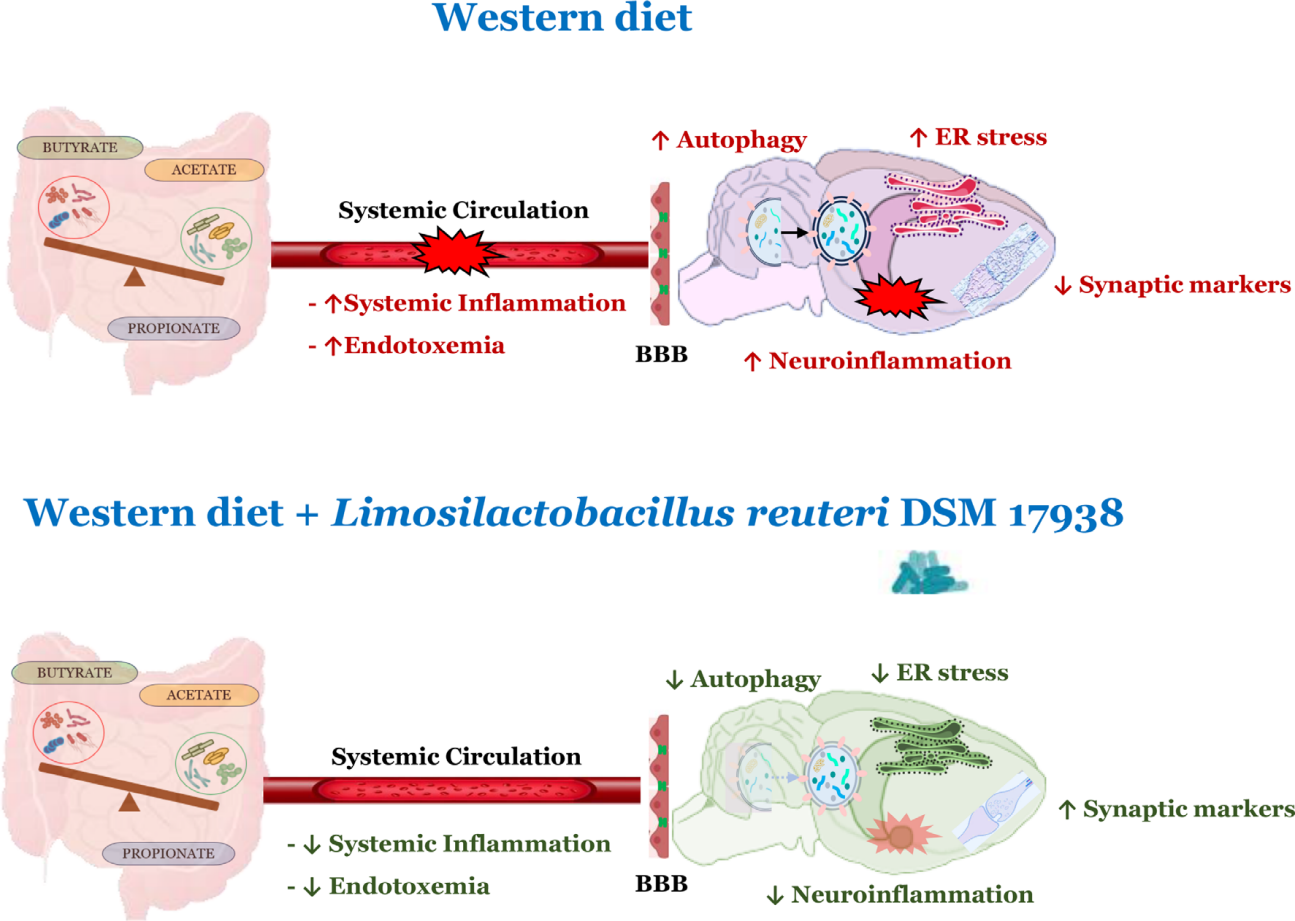
*Limosilactobacillus reuteri **DSM 17938*** impact on brain health of high fat fructose fed rats. Systemic and hippocampus alterations following high fat fructose diet (up) and high fat fructose diet plus *L. reuteri* supplementation (bottom). The up arrows indicate increased parameters, and the down arrows indicate reduced parameters. BBB, blood–brain barrier; ER, endoplasmic reticulum.

In conclusion, this study provides an overview of the complex interplay between nutrition and the brain, highlighting a different point of view about the impact that diet and probiotics can exert on our health and wellbeing, particularly in the context of diet‐induced neuroinflammatory condition. Notably, we here show that western diet‐induced generation of systemic‐ and neuroinflammation, ER stress and autophagy, and synaptic alterations in rat hippocampus, can be prevented by *L. reuteri* administration, showing for the first time a neuroprotective role of this specific probiotic strain (Figure 8). On this basis, it can be envisioned that therapeutic strategies based on the use of *L. reuteri* DSM17938 might be beneficial in addressing and/or reversing metabolic syndrome‐mediated brain dysfunctions and cognitive decline.

## AUTHOR CONTRIBUTIONS

Conceptualization: Arianna Mazzoli, Maria Stefania Spagnuolo, Susanna Iossa, Luisa Cigliano. Investigation: Arianna Mazzoli, Maria Stefania Spagnuolo, Francesca De Palma, Natasha Petecca, Angela Di Porzio, Valentina Barrella, Rosanna Culurciello, Antonio Dario Troise, Sabrina De Pascale, Gianluigi Mauriello. Formal Analysis: Arianna Mazzoli, Maria Stefania Spagnuolo, Antonio Dario Troise, Sabrina De Pascale, Andrea Scaloni, Susanna Iossa, Luisa Cigliano. Supervision: Arianna Mazzoli, Maria Stefania Spagnuolo, Susanna Iossa, Luisa Cigliano. Resources: Gianluigi Mauriello. Funding acquisition: Maria Stefania Spagnuolo, Andrea Scaloni, Luisa Cigliano. Writing original draft: Arianna Mazzoli, Maria Stefania Spagnuolo, Susanna Iossa, Luisa Cigliano. Writing‐review and editing: Arianna Mazzoli, Maria Stefania Spagnuolo, Antonio Dario Troise, Andrea Scaloni, Gianluigi Mauriello, Susanna Iossa, Luisa Cigliano. All authors have read and agreed to the published version of the manuscript.

## FUNDING INFORMATION

This work was supported by grants from: i) University of Naples Federico II ‐ Ricerca Dip 2021/2022 to LC; ii) Italian National Research Council for the project “Nutrizione, Alimentazione ed Invecchiamento Attivo (NUTRAGE)” (FOE 2021‐2022) to MSS and ADT; iii) the National Recovery and Resilience Plan, mission 4, component 2, investment 1.3, call n. 341/2022 of the Italian Ministry of University and Research funded by the European Union ‐ NextGenerationEU for the project “ON Foods ‐ Research and innovation network on food and nutrition Sustainability, Safety and Security ‐ Working ON Foods”, project PE00000003, concession decree n. 1550/2022, CUP E63C22002030007 (Unina), CUP B83C22004790001 (CNR); iv) National Recovery and Resilience Plan, mission 4, Component C2, funded by the European Union – NextGenerationEU, PRIN 2022PNRR (P2022LTFLR) to LC.

## CONFLICT OF INTEREST STATEMENT

The authors have no competing interests to disclose.

## Supporting information


**DATA S1:** Supporting Information.

## Data Availability

The data that support the findings of this study are available from the corresponding author upon reasonable request.
