# Supplementary material for: Limosilactobacillus reuteri DSM 17938 relieves inflammation, endoplasmic reticulum stress, and autophagy in hippocampus of western diet‐fed rats by modulation of systemic inflammation
Source: Biofactors. 2024 May 27;50(6):1236–50. doi: 10.1002/biof.2082 (PMC11627471; doi:10.1002/biof.2082)
Supplement: Supplementary file 1 — DATA S1: Supporting Information. [file BIOF-50-1236-s001.docx]

***Limosilactobacillus reuteri* DSM 17938 relieves inflammation, endoplasmic reticulum stress, and autophagy in hippocampus of western diet-fed rats by modulation of systemic inflammation**

Arianna Mazzoli ^1^, Maria Stefania Spagnuolo ^2^, Francesca De Palma ^1^, Natasha Petecca ^1^, Angela Di Porzio ^1^, Valentina Barrella ^1^, Antonio Dario Troise ^2^, Rosanna Culurciello ^1^, Sabrina De Pascale ^2^, Andrea Scaloni ^2^, Gianluigi Mauriello ^2^, Susanna Iossa ^1,4,5^, Luisa Cigliano ^1,5*^

^1^ Department of Biology, University of Naples Federico II, Complesso Universitario Monte S. Angelo, Edificio 7, Via Cintia, 80126 Naples, Italy.

^2^ Institute for the Animal Production System in the Mediterranean Environment, National Research Council, Piazzale Enrico Fermi 1, 80055 Portici, Italy.

^3^ Department of Agricultural Sciences, University of Naples Federico II, via Università 100, 80055 Portici, Italy

^4^ NBFC, National Biodiversity Future Center, Palermo 90133, Italy

^5^ Task Force on Microbiome Studies, University of Naples Federico II, Italy.

Arianna Mazzoli and Maria Stefania Spagnuolo contributed equally to this study.

* Correspondence

Luisa Cigliano, Department of Biology, University of Naples Federico II, Complesso Universitario Monte S. Angelo, Edificio 7, Via Cintia - I-80126 Napoli, Italy.

E-mail: luisa.cigliano@unina.it, phone number: +39-081-2535244; ORCID https://orcid.org/0000-0002-5491-9659

**Supplementary Table 1.** Diet composition

| Component | Composition g/1000 g | |
| --- | --- | --- |
|  | **Low Fat** | **Western diet** |
| Standard Chow^a^ | 395.3 | 231.5 |
| Sunflower oil | 19.3 | 19.3 |
| Casein | 59.7 | 133.3 |
| Water | 175.7 | 175.4 |
| AIN-93 Mineral mix | 11.4 | 11.4 |
| AIN-93 Vitamin mix | 3.2 | 3.2 |
| Choline | 0.7 | 0.7 |
| Methionine | 0.9 | 0.9 |
| Cornstarch | 333.8 | 0 |
| Butter | 0 | 129.8 |
| Fructose | 0 | 294.6 |
| Energy content and composition |  |  |
| ME content, kJ/g^b^ | 11.2 | 14.9 |
| Lipids, J/100 J | 10.5 | 39.3 |
| Proteins, J/100 J | 19.9 | 19.8 |
| Complex carbohydrates, J/100 J | 63.9 | 7.5 |
| Simple sugars, J/100 J | 5.7 | 33.4 |

^a^4RF21, Mucedola, Italy; ^b^Estimated by computation using values (kJ/g) for energy content as follows: Protein 16.736, lipid 37.656, and carbohydrate 16.736. ME = metabolizable energy; AIN= American Institute of Nutrition.

**Supplementary Table 2.** Dilutions of primary and secondary antibodies used for Western blotting.

|  | Primary Antibody | Secondary Antibody |
| --- | --- | --- |
| p-NFkB | Santa Cruz Biotechnology; 1:200 **^a^** | GAM-HRP IgG; 1:40,000 **^a^** |
| NFkB | Santa Cruz Biotechnology; 1:500 **^a^** | GAM-HRP IgG; 1:20,000 **^a^** |
| TLR4 | Sigma-Aldrich, 1:500 **^a^** | GAR-HRP IgG, 1:120,000 **^a^** |
| GFAP | Cell Signalling Technology; 1:1,000 **^b^** | GAR-HRP IgG, 1:250,000 **^b^** |
| Haptoglobin | Sigma-Aldrich, 1:750 **^a^** | GAR-HRP IgG, 1:220,000 **^b^** |
| ApoE | Merk-Millipore, 1:500 **^b^** | RAG-HRP IgG, 1:200,000 **^b^** |
| p-PERK | Cell Signalling Technology; 1:1,000 **^b^** | GAR-HRP IgG, 1:30,000 **^a^** |
| Lipocalin | Thermo Fisher Scientific, 1:300 **^a^** | RAG-HRP IgG, 1:120,000 **^a^** |
| PERK | Cell Signalling Technology; 1:1,000 **^b^** | GAR-HRP IgG, 1:50,000 **^a^** |
| CHOP | Cell Signalling Technology; 1:1,000 **^a^** | GAM-HRP IgG, 1:80,000 **^a^** |
| p-eif2α | Cell Signalling Technology; 1:1000 **^a^** | GAR-HRP IgG, 1:70,000 **^a^** |
| eif2α | Cell Signalling Technology; 1:1,000 **^a^** | GAM-HRP IgG, 1:60,000 **^a^** |
| p-GSK | Santa Cruz Biotechnology; 1:1,000 **^a^** | GAM-HRP IgG; 1:60,000 **^a^** |
| GSK | Santa Cruz Biotechnology; 1:500 **^a^** | GAM-HRP IgG; 1:30,000 **^a^** |
| Beclin | Santa Cruz Biotechnology; 1:500 **^a^** | GAM-HRP IgG; 1:50,000 **^a^** |
| LC3 II/I | Santa Cruz Biotechnology; 1:500 **^a^** | GAM-HRP IgG; 1:40,000 **^a^** |
| p62 | Cell Signalling Technology; 1:1000 **^a^** | GAR-HRP IgG; 1:50,000 **^a^** |
| Synaptophysin | Merk-Millipore; 1:100,000 **^a^** | GAR-HRP IgG; 1:45,000 **^b^** |
| Synaptotagmin | Cell Signalling Technology; 1:1000 **^a^** | GAR-HRP IgG; :350,000 **^b^** |
| PSD-95 | Cell Signalling Technology; 1:1000 **^a^** | GAR-HRP IgG; 1:40,000 **^b^** |
| Occludin | Thermo Fisher Scientific, 1:500 **^a^** | GAR-HRP IgG, 1:40,000 **^a^** |
| ZO-1 | Thermo Fisher Scientific, 1:500 **^a^** | GAR-HRP IgG, 1:100,000 **^a^** |
| IgG-HRP linked | Cell Signalling Technology; 1:12,000 **^d^** |  |
| β -Actin | Sigma-Aldrich; 1:1000 **^c^** | GAM-HRP IgG; 1:30,000 **^c^** |

GAR-HRP: Goat anti-rabbit horseradish peroxidase-conjugated IgG (Immunoreagents, Raleigh, NC, USA).

GAM-HRP: Goat anti-mouse horseradish peroxidase-conjugated IgG (Immunoreagents, Raleigh, NC, USA).

RAG-HRP IgG: Rabbit anti-goat horseradish peroxidase-conjugated IgG (Sigma Aldrich, Saint Louis, MO, USA).

T-TBS: 130 mM NaCl, 20 mM Tris-HCl, 0.05% Tween, pH 7.4.

^a^ T-TBS containing 2% w/v BSA.

^b^ T-TBS containing 1% v/v non-fat milk.

^c^ T-TBS containing 0.25% v/v non-fat milk.

^d^ T-TBS containing 5% v/v non-fat milk

**Supplementary Table 3.**

Elemental composition and retention time of the analytes quantified; in bold internal standard used for quantification, yield and recovery of the derivatization procedure. Mass measurement error (Error, Δ ppm) was calculated as the ratio between the difference of the experimental mass minus the theoretical (exact) mass and the theoretical (exact) mass, multiplied per one million. Each compound is intended as hydrazone derivative.

| **Compound Name** | **Elemental Composition** | **RT (min)** | ***m/z theoretical*** | ***m/z experimental*** | **Δ ppm** |
| --- | --- | --- | --- | --- | --- |
| acetic acid | C_8_H_9_N_3_O_3_ | 9.10 | 194.0571 | 194.0573 | 1.03 |
| propionic acid | C_9_H_11_N_3_O_3_ | 11.10 | 208.0728 | 208.0724 | -1.92 |
| butyric acid | C_10_H_13_N_3_O_3_ | 12.80 | 222.0884 | 222.0888 | 1.80 |
| **^13^C_2_-acetic acid** | ^13^C_2_C_6_H_9_N_3_O_3_ | 9.10 | 196.0638 | 196.0638 | 0.00 |
| **^13^C_3_-propionic acid** | ^13^C_3_C_6_H_11_N_3_O_3_ | 11.10 | 211.0828 | 211.0828 | 0.00 |
| **^13^C_4_-butyric acid** | ^13^C_4_C_6_H_11_N_3_O_3_ | 12.80 | 226.1018 | 226.1019 | 0.44 |

**Supplementary Figure 1. Ponceau staining of nitrocellulose membrane**


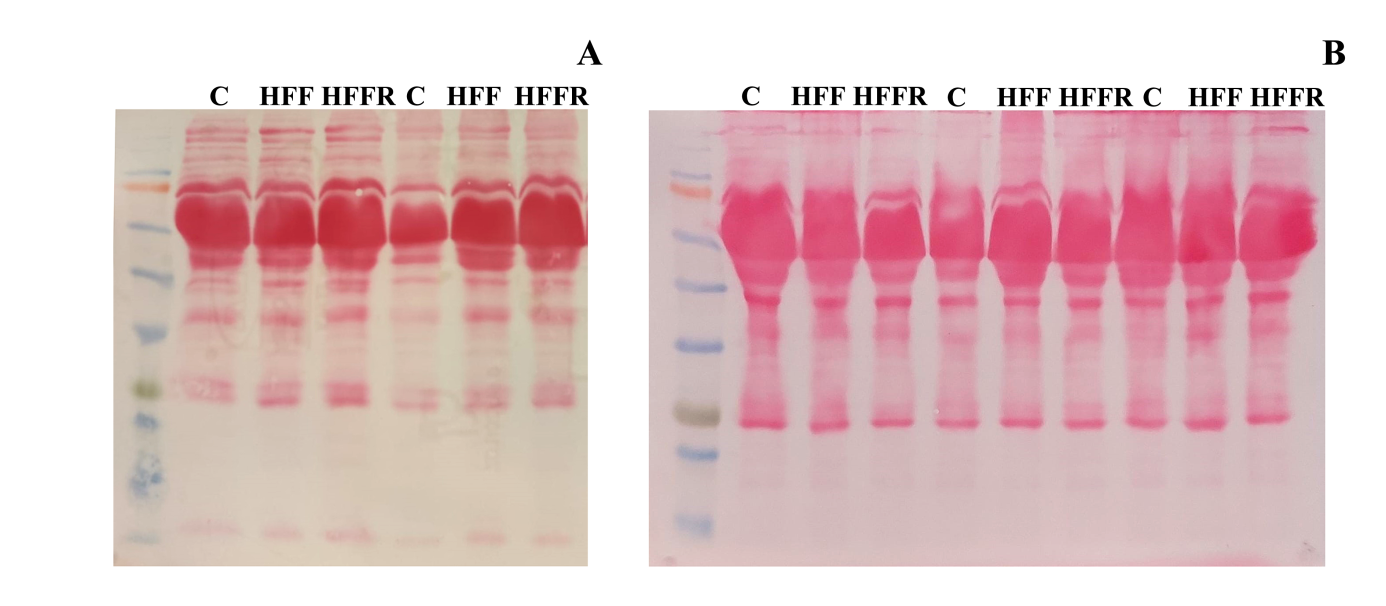


Plasma proteins (80 µg) from rats fed control diet (C), high fat fructose diet (HFF) or high fat fructose diet supplemented with *L. reuteri* (HFFR) were fractionated on 12.5 % polyacrylamide gels. After blotting, nitrocellulose membranes were stained with 0.1 % Ponceau S (solubilized in 5% Acetic Acid) to verify the effectiveness of transfer and to determine sample loading in each lane. Membranes were then washed, blocked and incubated with anti-Hpt IgG (A) or anti-lipocalin (B) as described in the text.


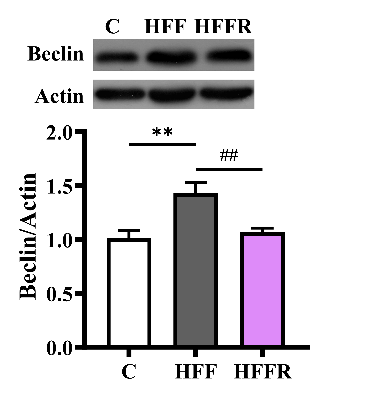
**Supplementary Figure 2. Beclin western blot**

Beclin protein content (with representative blots, normalized to controls) in hippocampus from rats fed control diet (C), high fat fructose diet (HFF) and high fat fructose diet supplemented with *L. reuteri* (HFFR). Values are the means ± SEM of 8 different rats. **p<0.01 compared to C rats; ## p<0.01 compared to HFF rats (One way ANOVA followed by Bonferroni post-test).
